# Acoustic immittance measures and middle ear assessment: Current practice by South African audiologists

**DOI:** 10.4102/sajcd.v68i1.818

**Published:** 2021-06-07

**Authors:** Ben Sebothoma, Katijah Khoza-Shangase

**Affiliations:** 1 Department of Speech Pathology and Audiology, Faculty of Humanities, University of the Witwatersrand, Johannesburg, South Africa

**Keywords:** acoustic immittance, audiological practice, middle ear function, South Africa, audiology, tympanometry

## Abstract

**Background:**

Limited research exists regarding South African audiologists’ practice with acoustic immittance. This study was part of a bigger study titled ‘Wideband acoustic immittance in adults living with human immunodeficiency virus’.

**Objectives:**

The purpose of the study was to explore current practice of South African audiologists regarding acoustic immittance measures, and to explore their perceived knowledge and views on acoustic immittance advancements.

**Method:**

A quantitative survey with a cross sectional design was employed. An electronic questionnaire was distributed to participants via professional associations of audiologists. Data was analysed through descriptive and inferential statistics.

**Results:**

Most audiologists worked in private practice and conducted tympanometry with 226Hz probe tone and ipsilateral acoustic reflexes. There was no association between clinical setting, levels of qualification, and the use of tympanometry. None of the participants included multifrequency and multicomponent tympanometry (MFT) and/or wideband acoustic immittance (WAI) in their test battery. Most of the participants were not familiar with MFT and WAI. Familiarity with MFT and WAI were only associated with the level of qualification. Limited training and lack of equipment were major barriers to conducting some of the acoustic immittance measures. Most participants believed that they would include MFT and/or WAI in their test battery if they had access to the equipment.

**Conclusion:**

Current findings raise training and clinical implications for the South African audiologists, including training institutions. These findings provide motivation for strategic resource allocation, planning and distribution of audiology clinics in the country if positive preventive audiology outcomes are to be achieved.

## Background

Acoustic immittance measures consist of tympanometry, acoustic reflex threshold (ART) and acoustic reflex decay (ARD) (Martin & Clark, 2015). These measures are designed to assess middle ear function (Hunter & Shahnaz, 2014) and to provide a differential diagnosis for various disorders along the auditory pathway (British Society of Audiology [BSA], 2013). As a result, acoustic immittance measures form part of the standard audiological test battery for both adults and children (American Academy of Audiology [AAA], 2012; Health Profession Council of South Africa [HPCSA], 2012). Despite the established value of this measure, there seem to be discrepancies in the literature about its use in clinical practice (Emanuel, Henson & Knapp, 2012). In low- and middle-income countries (LMICs), including South Africa, there is limited research on the practice of audiologists regarding acoustic immittance. This research is particularly important where preventive audiology is arguably a part of the re-engineered primary healthcare strategy adopted by the South African government and where the main study ‘Wideband acoustic immittance in adults living with human immunodeficiency virus’ is located.

MacDonald and Green (2001) showed that 91% of their participants in Canada always include tympanometry with 226 Hz probe tone in their basic audiological test battery and 77% always include both ipsilateral and contralateral reflex threshold testing. However, a survey conducted in the United States of America (USA) by Emanuel et al. (2012) found that only 61% of their participants use tympanometry with 226 Hz probe tone routinely. Both these studies found that over 70% of their participants never use multi-frequency and multicomponent tympanometry in their clinical practice. These studies support an earlier study by Martin, Champlin and Chambers (1998) who found that approximately 10% of the participants reported using multi-frequency and multicomponent tympanometry in the USA. Overall, evidence indicates that, firstly, it is not known whether there is any change in the use of acoustic immittance in clinical settings globally. Secondly, available studies on acoustic immittance measures were mainly conducted in Western high-income countries, with limited research from LMICs. Finally, none of these studies have investigated the use of wideband acoustic immittance (WAI).

Variability in acoustic immittance practices among audiologists is concerning, given the high prevalence and impact of middle ear pathologies in both adults and children. The World Health Organization (2018) reports that middle ear pathologies affect over 700 million people per annum globally, with LAMI countries being the most affected (DeAntonio et al., 2016) because of multiple risk factors such as human immunodeficiency virus (HIV), lack of awareness and knowledge (Joubert, Sebothoma, & Kgare, 2017; Sebothoma & Khoza-Shangase, 2020). Whilst middle ear pathologies can be treated medically (Rosenfeld et al., 2016), prolonged or late identification and intervention may lead to further complications (Kolo et al., 2012), which are difficult to treat and may require specialised services that are scarce in LMICs (Mulwafu et al., 2017). Therefore, it is crucial that audiologists include acoustic immittance measures in their basic audiological test battery in order to identify early signs of middle ear pathologies, for preventive ear and hearing care to occur. In South Africa, the standard audiological test battery requires inclusion of acoustic immittance for identification of middle ear pathologies and making referrals accordingly.

Literature has suggested that acoustic immittance measures provide important clinical information about the mechanical characteristics of the middle ear system (Kramer & Brown, 2019). Bess and Humes (2008) reported that the mechanical properties of the middle ear system are often affected by the presence of middle ear pathologies. Therefore, the use of acoustic immittance allows the audiologists and other ear and hearing health professionals to objectively identify the abnormalities within the middle ear system. As a result, the inclusion of acoustic immittance measures in the test battery is recommended (BSA, 2013). Although acoustic immittance forms part of the standard audiological test battery, tympanometry with 226 Hz probe tone remains the most routinely used measure of middle ear assessment in audiological clinics (Martin & Clark, 2015). Emerging research has indicated that tympanometry with 226 Hz probe tone has poor sensitivity and specificity in the identification of middle ear pathologies (Kaf, 2011; Sebothoma & Khoza-Shangase, 2018). The continuous use of tympanometry with 226 Hz probe tone results from the ease with which these measures can be performed and obtained and results can be interpreted (Hunter & Shahnaz, 2014). It is therefore clear that audiologists, especially those working in resource-constrained contexts, need to move towards including more accurate measures of middle ear pathologies to improve early identification and intervention and to reduce costs associated with untreated middle ear pathologies.

Multi-frequency and multicomponent tympanometry and WAI have been shown to be more accurate in detecting middle ear pathologies than the traditional tympanometry with 226 Hz probe (Shahnaz & Polka, 1997). The superiority of these advanced acoustic immittance measures is reported to be a result of their clinical parameters, which include the ability to measure middle ear function over a wide range of frequencies (Sanford et al., 2013) and detect resonant frequency (RF) (Iacovou et al., 2013). These clinical parameters allow such measures to accurately identify early signs of various middle ear pathologies (Ibraheem, 2014) and predict conductive hearing loss (Keefe et al., 2012). Assessing whether audiologists include advanced acoustic measures such as multi-frequency and multicomponent tympanometry and WAI has implications for clinical practice and resource allocation, particularly in LMICs. These implications include early identification and timely interventions for middle ear pathologies, as part of the preventive healthcare approach that South Africa embraces.

Based on the information presented above, the purpose of this study was to explore the current practices of South African audiologists regarding acoustic immittance and to explore their perceived knowledge and views regarding advanced measures such as multi-frequency multicomponent tympanometry and WAI.

The specific objectives of the study were to:

- determine the acoustic immittance measures conducted by South African audiologists in clinical practice
- describe facilitators and barriers to the use of acoustic immittance in clinical practice
- describe audiologists’ familiarity with advanced acoustic immittance measures.

## Method

This was a quantitative survey research study with a cross-sectional design (Leedy & Ormrod, 2013). This design was deemed appropriate as the research aimed to explore current acoustic immittance practices by South African audiologists. A web-based survey was employed as it allowed the researchers to gather data from audiologists across South Africa (Khoza-Shangase & Masondo, 2020). Wright (2005) reported that survey research provides access to participants from distant locations.

### Data collection method

Data collection for this study started with survey development. The questionnaire was developed based on previous surveys that were conducted in Western countries such as Canada and the USA (Emanuel et al., 2012; MacDonald & Green, 2001). However, items for this study were modified to suit the current study and the South African context. The survey questionnaire was reviewed by two qualified audiologists who were selected based on their routine use of and expertise in acoustic immittance measures. They provided feedback on clarity of the questions and content, that is, relevance of the items for the study. A pilot study was then conducted with five audiologists who met the inclusion criteria to determine the feasibility of the questionnaire and to obtain additional inputs about the survey. The final version of the questionnaire was uploaded onto SurveyMonkey, which was used to create the survey.

### Participants

A non-probability purposive sampling method was used to recruit potential participants who met the inclusion criteria (Leedy & Ormrod, 2013). Participants had to be practising audiologists registered with the HPCSA. Participants holding a dual degree in speech language pathology and audiology (SLP/A) but working primarily as speech language pathologists (SLPs) were excluded from the study. The completed survey questionnaire was sent to two professional associations (South African Association of Audiologists [SAAA] and South African Speech Language Hearing Association [SASLHA]) for distribution. The South African Speech Language Hearing Association distributed the survey link by emailing it to all their members, but SAAA distributed the survey link on their social media pages such as Facebook and WhatsApp. A 3-month cut-off time was set for participants to respond to the survey. Reminders were sent to potential participants by SAAA and SALHA.

### Data analysis

Data were analysed using the Statistical Package for the Social Sciences (SPSS) version 25. Both descriptive and inferential statistics were employed (Ali & Bhaskar, 2016). Pearson’s chi-squared test was used to determine the association between variables of interest: level of education, clinical setting and the use of tympanometry with 226 Hz probe tone. The *p*-value for statistical significance for this study was set at *p* ≤ 0.05.

### Reliability and validity

To determine content and face validity, following the review by two experienced audiologists, a pilot study was conducted where the questionnaire was sent via SurveyMonkey using the same procedure as described for the main study. The audiologists who formed part of the pilot study were asked to comment and provide feedback on the content and to determine whether the content of the questionnaire is in line with the objective of the study (Satake, 2015). Findings at this stage of the study revealed that the content of the questionnaire was appropriate for the study and captured what was being investigated. Furthermore, the pilot study also revealed that the questionnaire could be completed in approximately 10 min. It should be noted, however, that validity was compromised by the very nature of online surveys, where results might not have been a true reflection of assessment practices.

### Ethical considerations

All procedures in this study adhered to the World Medical Association (WMA) Declaration of Helsinki (Millum, Wendler & Emanuel, 2013) ethical guidelines. Ethical clearance was obtained from the Human Research Ethics committee (HREC) (non-medical) of the University of the Witwatersrand, Johannesburg, South Africa (Protocol Number: H19/02/30). Participants gave written informed consent by clicking the button on the SurveyMonkey after reading and understanding the study information sheet.

## Results

**FIGURE 1:**
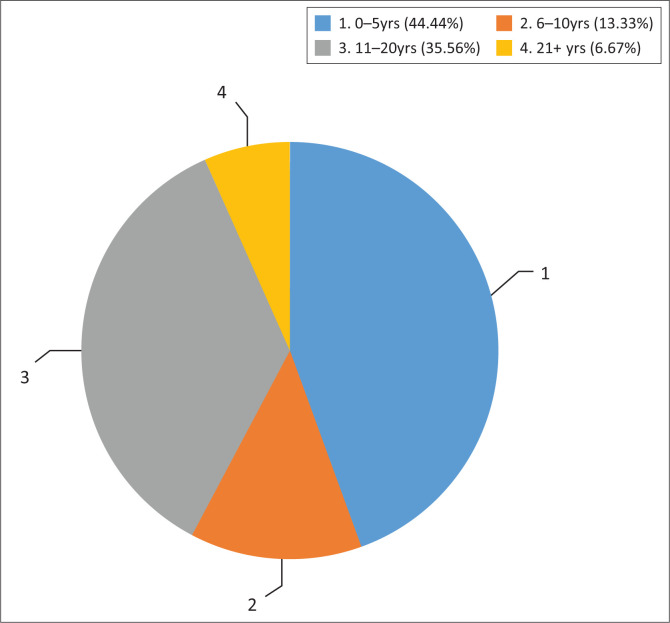
Number of years practising as audiologists.

**FIGURE 2:**
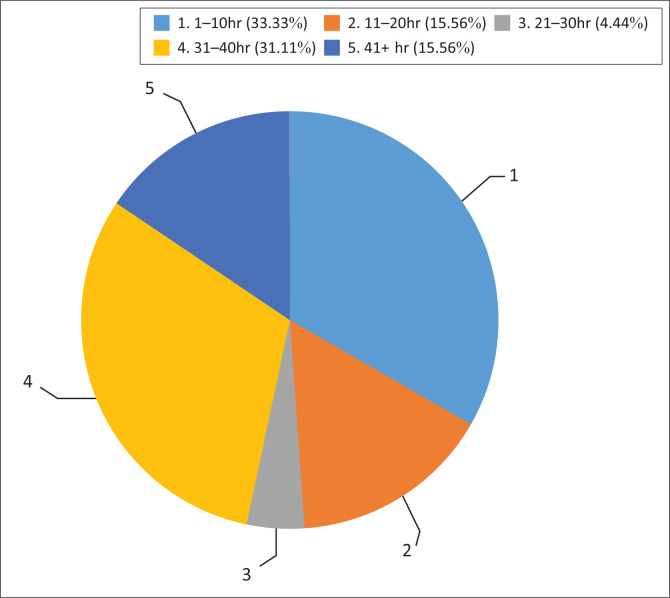
Number of hours spent on clinical practice tasks on a weekly basis.

Forty-five audiologists responded to the survey. Over half (53.3%) of the participants were employed in public sector facilities, such as public hospitals, primary healthcare sectors and schools, whilst 40% (*n* = 18) of the participants worked in private practice. Very few participants worked in university clinics (2.2%; *n* = 1) and non-profit or non-governmental organisations (4.4%; *n* = 2). Participants had various levels of experience and numbers of hours they provided clinical audiological services per week. Figure 1 summarises the number of years that participants had been in clinical practice, whilst Figure 2 provides the average number of hours that participants spent providing clinical audiological services in their respective settings. Most of the participants (64.4%; *n* = 29) hold an undergraduate degree, which is a standard practice in South Africa. An undergraduate degree is sufficient for registration to practice.

### Conventional acoustic immittance measures

Table 1 summarises the frequency with which participants included various acoustic immittance measures, whilst Table 2 shows different age groups assessed by various acoustic immittance measures. A majority of participants (68.9%; *n* = 31) indicated that they always include tympanometry with 226 Hz in their audiological test battery, with very few participants indicating that they never (2.2%; *n* = 1) include it or rarely (2.2%; *n* = 1) include it in their audiological test battery. Of those who include tympanometry with 226 Hz probe tone, 57.8% (*n* = 26) reported that they use it to assess patients who are over 6 months old, whilst 40% (*n* = 18) of the participants use tympanometry with 226 Hz probe tone with all patients.

**TABLE 1:** Use of various acoustic immittance measures.

Type of measure	Number of participants (%)	Frequency of use
Tympanometry with 226 Hz probe tone	84.5	Always
	11.1	Sometimes
	-	-
	2.2	Never
	2.2	Rarely
Tympanometry with 1000 Hz probe tone	40	Always
	24.4	Sometimes
	-	-
	22.2	Never
	13.3	Rarely
Acoustic reflex threshold	73.3	Always
	11.1	Sometimes
	-	-
	2.2	Never
	13.3	Rarely
Acoustic reflex decay	6.6	Always
	13.3	Sometimes
	-	-
	44.4	Never
	35.6	Rarely

**TABLE 2:** Age of patients assessed by various acoustic immittance measures.

Type of measure	Number of participants (%)	Age of patients assessed
Tympanometry with 226 Hz probe tone	58	˃ 6 months
	40	All patients
Tympanometry with 1000 Hz probe tone	64	≤ 0–6 months
	18	All patients

Pearson’s chi-squared test indicates that there was no statistically significant association between the levels of participant qualifications (e.g. Bachelor of Science [BSc] or Master of Arts [MA]) and the use of tympanometry with 226 Hz probe tone (*p* = 1.000). There was also no statistically significant association between working in any setting (e.g. public or private) and using tympanometry with 226 Hz probe tone (*p* = 0.116). Tympanometry with 1000 Hz probe tone was used infrequently, with only 26.7% (*n* = 12) of participants always including it in their audiological test battery. A majority of participants (64.4%; *n* = 29) used tympanometry with 226 Hz probe tone only with children aged 0–6 months. Just below half (48.9%; *n* = 22) of the participants stated that they always include ART as part of their audiological test battery, with a negligible number (2%; *n* = 1) who never include ART. There was no association between level of education (*p* = 0.84) or clinical setting (0.95) and the use of ART.

Of those who included ART, 71.1% (*n* = 32) of the participants indicated that they only included ipsilateral ART. However, there was no association between level of education and the type of ART included (*p* = 0.068). Of this sample, 33.3% (*n* = 15) of the participants indicated that their equipment did not provide both the ipsilateral and contralateral ART tests, whilst others cited lack of time (24%) in conducting both ipsilateral and contralateral testing. Almost all participants (80%) never included ARD in their audiological test battery. Chi-squared test also revealed that there was no association between the level of education (*p* = 0.8796) or clinical setting (*p* = 0.315) and the inclusion of ARD.

### Advanced acoustic immittance measures

A majority of participants never included multi-frequency and multicomponent tympanometry (82.2%; *n* = 37) and/or WAI (73.3%; *n* = 33) in their audiological test battery. Those who did not include multi-frequency and multicomponent tympanometry and/or WAI cited the lack of equipment for multi-frequency and multicomponent tympanometry (26.7%; *n* = 12) and WAI (33.3%; *n* = 15) and training for both tests (51.1% and 44.4%) as the main reasons.

Regarding familiarity, only 2.2% (*n* = 1) of the participants were familiar with multi-frequency and multicomponent tympanometry and WAI. Further analysis indicated that familiarity with multi-frequency and multicomponent tympanometry (*p* = 0.0004) and WAI (*p* = 0.0273) was associated with participants’ levels of education. However, the familiarity with multi-frequency multicomponent tympanometry (*p* = 0.913) and WAI (*p* = 0.994) was not associated with the type of clinical setting (e.g. private vs. public). Participants were also asked if they would include multi-frequency and multicomponent tympanometry and/or WAI if they had equipment; 57.8% (*n* = 26) and 35.6% (*n* = 16) confirmed this and 37.8% (*n* = 17) and 60% (*n* = 27) were unsure.

## Discussion

The aims of this study were to explore the current practice of South African audiologists regarding acoustic immittance measures and to describe their familiarity with advanced acoustic immittance measures. The results reveal that 68.9% (*n* = 31) of the participants include tympanometry with 226 Hz probe tone in their audiological test battery. These results are consistent with previous studies conducted in Canada and USA, which established that audiologists always include tympanometry with 226 Hz probe tone in their audiological test battery (Emanuel et al., 2012; MacDonald & Green, 2001).

A higher number of participants’ frequent inclusion of tympanometry with 226 Hz probe, and less of other tympanometry, could be attributed to its availability and the ease with which the test can be performed and its results can be interpreted (Erkkola-Anttinen et al., 2014). For example, only 2% (*n* = 1) of the participants indicated not having equipment. Those who did not always include tympanometry with 226 Hz probe tone cited ‘other’ reasons other than availability. A high number of participants indicated that they did not always include tympanometry with 1000 Hz probe tone; instead, they used tympanometry with 226 Hz probe tone with all patients. These findings are inconsistent with the survey conducted by Meyer, Swanepoel and Le Roux (2014), which found that most of the participants included high-frequency tympanometry. The difference between these studies can be attributed to different sample sizes and settings. The latter study sampled participants only from the private sector, whilst the current study included participants from various clinical settings. Therefore, the differences are not surprising given that those in the private sector are known to have more resources than those in the public sector (Teixeira & Joubert, 2014).

Despite the differences in the findings, not including tympanometry with 1000 Hz probe tone raises implications for early identification and intervention of middle ear pathologies in children under the age of 6 months. Kramer and Brown (2019) reported that tympanometry with 226 Hz is invalid in identifying middle ear pathologies in young children under the age of 6 months, perhaps because of the residual mesenchyme in the middle ear (Shanks & Shohet, 2009). Park et al. (2015) found the sensitivity of tympanometry with 226 Hz prone tone in identifying middle ear pathologies in children under the age of 6 months to be poor, ranging from 0% to 6.6%. However, the continuous use of tympanometry with 226 Hz probe tone in patients of all ages affects early detection (HPCSA, 2018). Furthermore, given the scarcity of resources to deal with severe middle ear pathologies (Fagan & Jacobs, 2009), the use of tympanometry with 226 Hz probe in all patients might have long-term negative consequences.

In terms of ART and ARD, almost 50% of the participants always included ART, whilst a majority (80%) never included ARD. Of those who include ART, a majority (71%; *n* = 32) only include ipsilateral ART because their equipment only provides this option and does not provide an option for contralateral ART. These findings are consistent with the results of a study by Emanuel et al. (2012) who found that a majority of their participants always included ART, but few included ARD in their assessments. Unlike in the current study, a majority of participants (91%) in the study by Emanuel et al. (2012) used both ipsilateral and contralateral ART. Given that ART and ARD are essential for differentiating cochlear from retrocochlear disorders (Kramer & Brown, 2019), it is concerning that some ART and ARD are not consistently conducted. Some participants (24.4%; *n* = 11) stated that they had no time to conduct both the ipsilateral and contralateral ART. This may be because of time management, scheduling of appointments and workload challenges in their practices.

A majority of participants in our study indicated that they did not include multi-frequency and multicomponent tympanometry and WAI and were not familiar with these tests. A higher number of participants stated that they were not trained in the use of these tests and did not have the required equipment. Tympanometry with a wide range of frequencies such as WAI is not yet widespread in clinical practice (Kramer & Brown, 2019), particularly in developing countries because of the lack of normative values (Sebothoma et al., submitted). Several studies have already indicated that tympanometry with a wide range of frequencies have higher sensitivity and specificity in identifying middle ear pathologies (Kaf, 2011; Shahnaz et al., 2009); therefore, academic institutions in South Africa need to teach and update audiology students and graduates – through seminars and workshops – advancements in audiology. It was comforting to see that over 80% of the participants were amenable to using multi-frequency and multicomponent tympanometry and WAI if they were to receive the equipment.

In an attempt to establish if there were any trends in the data that could explain the outcomes in terms of current practice, factors such as the level of qualification, sector of employment and length of employment were carefully qualitatively scrutinised. Of these factors potentially influencing practice, only private practice placement seemed to have a positive influence on current practice, possibly because of the availability of resources that are otherwise not widely available in state institutions that are fraught with resource constraints. The level of education of the participants appeared to have no influence in current findings. This was expected as middle ear assessment forms part of the HPCSA minimum standards for undergraduate qualification in audiology in South Africa, with postgraduate training comprising research only, research report and research coursework, with no clinical training at postgraduate level (Khoza-Shangase & Masondo, 2020).

## Study limitations

Whilst this study provided information about practices of South African audiologists regarding acoustic immittance, there are methodological limitations that need to be taken into consideration whilst interpreting the findings. Although the associations’ data basis used represents a large number of audiologists in the country, unlike any other, the recruitment method used in this study permitted access only to audiologists affiliated with these associations (SAAA and SASLHA) and those with Internet access to participate. This has resulted in a small sample size that is not necessarily a representative of a sample of the audiologists working in various sectors in South Africa, thus limiting the applicability in terms of guideline implementation and generalisation to the majority of the country’s practitioners. This limitation raises implications for future studies to explore the same question by including participants who are not associated with SAAA and SASLHA. For example, future studies should consider recruiting participants through the national forum as well, the National Black Speech Language Hearing Associations and other existing platforms to increase the representation of audiologists.

## Conclusion

The results of this study indicate that South African audiologists do not regularly conduct acoustic immittance measures, with a majority conducting only tympanometry with 226 Hz prone tone and ipsilateral ART regularly. The variance in practice is mainly attributed to the lack of resources and limited training at undergraduate level. It was also noted that most of the audiologists are not familiar with multi-frequency multicomponent tympanometry and WAI. Therefore, this study highlights the need for enhanced training and appropriate resource allocation where audiological services are provided. Educating both undergraduate and postgraduate students about the importance of including acoustic immittance in routine test battery and the various measures for specific circumstances is required. Furthermore, equipping South African audiologists with the knowledge of advanced acoustic immittance measures that are more effective in identifying middle ear pathologies, such as WAI, may keep them abreast with new developments in the field. All this may contribute positively to early identification and consequent early treatment of middle ear pathologies as part of preventive healthcare measures within this LAMI context, where a high burden of diseases that increase occurrences of middle ear disease, such as HIV or AIDS, exists.
